# Mating of unfed, engorged, and partially to fully gravid *Aedes aegypti* (Diptera: Culicidae) female mosquitoes in producing viable eggs

**DOI:** 10.1186/s13071-024-06433-z

**Published:** 2024-08-25

**Authors:** Muniaraj Mayilsamy, Surendiran Subramani, Rajamannar Veeramanoharan, Asifa Vijayakumar, Amuthalingam T. Asaithambi, Arthi Murugesan, Nandhakumar Selvaraj, Vijayakumar Balakrishnan, Paramasivan Rajaiah

**Affiliations:** ICMR-Vector Control Research Centre Field Station, No. 4 Sarojini Street, Chinna Chokkikulam, Madurai, 625002 Tamil Nadu India

**Keywords:** *Aedes aegypti*, Mating, Unfed, Fully fed, Gravid, Engorged, Viable eggs

## Abstract

**Background:**

Understanding the relationship between blood-feeding and mating is important in effectively managing the most well-adapted vector insect, *Aedes aegypti* (Linnaeus). Although extensive studies have investigated the behavioural aspects of *Aedes* such as blood-feeding, mating, and their relationship, several knowledge gaps still exist. Therefore, the present study was undertaken to determine the possibility of successful mating by unfed, engorged, and partially to fully gravid (up to 5 days after blood-feeding with fully developed eggs) female *Ae. aegypti* mosquitoes and production of viable eggs.

**Methods:**

Mating of sexually mature adult Aedes aegypti was allowed in three different ways. In control 1, the females were allowed to mate before taking blood meal, and in control 2, the females were not at all allowed to mate. In the experiment, the females were separated into six categories, viz. D-0 to D-5. In D-0, the females were allowed to mate immediately after the bloodmeal and, in D-1, the females were allowed to mate on the first day of blood feeding, likewise, the females of D-2, D-3, D-4 and D-5 were allowed to mate on 2nd, 3rd, 4th and 5th day of blood feeding. Ovitrap was uniformly kept on the 4th day of blood feeding for the cages D-0 to D-3 for 1 h and then removed and for the cages D-4, and D-5, the ovitrap was kept on 4th and 5th day of blood feeding for 1h immediately after mating. The total number of eggs and the total number of hatching were counted. In the subsequent days, the entire experiment was replicated two times with different cohorts of mosquitoes, and the mean value of three experiments was used to draw Excel bars with 5% error bars and also for the statistical analysis.

**Results:**

It was found that mating just before oviposition was sufficient to produce 1581 eggs (70% compared with control) and fertilize 1369 eggs (85% compared with total eggs laid), which is far higher than the 676 non-hatching (unfertilized) eggs (30%) laid by unmated females. Although mating is not essential for producing eggs, our study shows that even brief exposure to the semen and seminal fluids greatly enhances the oviposition and hatching efficiency, even if the mating occurs just before oviposition. However, those females mating before blood-feeding and those mating after blood-feeding produced 2266 and 2128 eggs, with hatching rates of 96.78% and 95.54%, respectively. Hence, the retention time of seminal fluid in the female seems to influence the number of eggs laid and the number of eggs hatched.

**Conclusions:**

In general, mating is possible in *Ae. aegypti* even minutes before oviposition and is sufficient to produce a greater number of viable eggs.

**Graphical Abstract:**

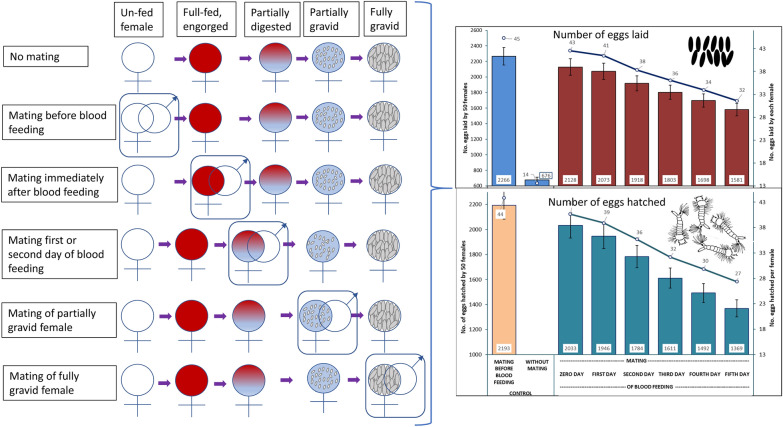

## Background

The mosquitoes *Aedes aegypti* (L.) and *Aedes albopictus* (Skuse) are a major public health concern, responsible for the transmission of viruses causing dengue [1], Zika [2], chikungunya [3], and yellow fever [4]. Their development in tropical and subtropical regions is intrinsically linked to human dwellings and activities such as rapid urbanization, which offer adequate larval growing habitats and the blood meal hosts required for their reproduction [5]. Among the diseases transmitted by *Ae. aegypti* and *Ae. albopictus, *dengue alone threatens nearly half of the world’s population across 129 countries, with approximately 96 million clinical cases and 40,000 deaths every year [6]. Despite vaccine availability since the 1930s, yellow fever remains a major threat to the people living in Africa and in South and Central America [7]. Two vaccine candidates (CYD-TDV [Dengvaxia, Sanofi Pasteur] and TAK-003 [Qdenga, Takeda Pharmaceuticals]) have been licensed for dengue, and a third candidate (Butantan-DV) has recently passed a phase 3 trial in Brazil [8], but none of them is currently available for public use in the affected countries. As there are no effective drugs available for dengue and similar *Aedes*-transmitted diseases such as yellow fever, the treatment relies solely on symptomatic supportive therapy [9]. Although vector control strategies using appropriate larvicides and adulticides play an important role in dengue control programs [10–12], dengue control efforts are complicated by the development of resistance in *Aedes* mosquito populations to the commonly used insecticides [13]. For the transmission of the dengue virus, or any other arboviruses, an infected host needs to be bitten by a female mosquito for a blood meal to develop her first batch of eggs, and subsequent bites for laying second or further cycles of eggs after a considerable period [14]. Hence, as mating and blood-feeding are connected with host-seeking and disease transmission, every aspect of the crosstalk between male and female *Aedes* mosquitoes needs to be investigated. Immediately after emergence, the female *Ae. aegypti* are unreceptive to mating [15], and males are also unable to mate until their abdominal terminalia rotate, which takes approximately 24 h. Once the female initiates flight, the sexually mature males will attempt to mate by engaging their genitalia [16].

Although many researchers have conducted extensive studies on the behavioural aspects of *Aedes*, such as blood-feeding, mating, and their relationship [17–22], existing knowledge gaps have yet to be explored [23]. A report that blood-fed *Aedes* females were generally inactive and had lower chances for copulation [24, 25] contradicts reports that the rate of mating after blood-feeding was high [26, 27]. A very recent report by Ramirez-Sanchez [28] connects female *Ae. aegypti* age to blood-feeding, insemination, sperm storage, and fecundity. In female *Ae. aegypti*, mating results in decreased sexual receptivity [29, 30] and changes in gene expression in female reproductive tissues [31], including the long-term sperm storage sac [29], the spermatheca [32, 33], which produces products essential for optimal mosquito fertility [33, 34]. It is not known whether semen holding time or retention time in spermathecae is short when mating occurs just before oviposition. Irrespective of mating, a fully fed, engorged female has to spend 4 to 5 days digesting the blood meal and subsequent development of eggs. Hence, an unmated but blood-fed female has an enormous length of time, from taking a blood meal to oviposition, to achieve successful copulation. However, the success rate of laying viable eggs if the female encounters a male during the time from immediately after taking the blood meal to just before oviposition is not known. Although female *Ae. aegypti* require a blood meal to develop their eggs, the age at which they would have their first blood meal is unclear [35]. Reports are also not clear on whether females mate before or after a blood meal [15].

Closing this knowledge gap in mating versus blood-feeding is important, particularly for vectors such as *Aedes*, in which re-mating of females is rare [36–38]. As mating behaviour is one of the most understudied aspects of mosquito biology, the present study will provide some important pieces of information on the reproductive biology of *Aedes* mosquitoes to better understand the process. Therefore, this study was undertaken to determine the possibility of successful mating and production of a total number of eggs and total number of viable eggs by unfed, engorged, and partially to fully gravid (up to 5 days after blood-feeding with fully developed eggs) female *Ae. aegypti* mosquitoes.

## Methods

### *Aedes aegypti* collection and colony maintenance

Adult *Ae. aegypti* mosquitoes were collected from Madurai (latitude: 9°56′10.97″ N, longitude: 78°08′09.33″ E; elevation: 140 m) in Tamil Nadu State, India, by indoor resting collection and stabilized in the mosquito colony of this centre (Indian Council of Medical Research-Vector Control Research Centre [ICMR-VCRC] Field Station, Madurai, Tamil Nadu, India) for three generations before being used in the experiment. The standard operating procedure as described by Zheng et al. [39] for mass rearing, egg storage, and hatching of *Ae. aegypti* was followed, with minor modifications. Briefly, the adult *Ae. aegypti* populations were maintained in 60 × 60 × 60 cm cages (BugDorm-6610, India) at room temperature (RT) of 27 ± 1 °C and relative humidity (RH) of 85 ± 5% with a photoperiod of 12:12 h (L:D). A cotton pad soaked in 10% glucose along with wet raisins was given in each cage as a sugar source for the adult mosquitoes. The glucose pad and wet raisins were changed at an interval of 2 days to avoid fungal growth. After 2 days of glucose feeding, the females were allowed to take a chicken blood meal and then were allowed to rest for 3 days before being placed for oviposition. A filter paper (Whatman) strip was kept half-soaked in each half-water-filled bowl for oviposition.

### Developing adults for mating experiment

Approximately 1500 freshly laid *Ae. aegypti* eggs were kept for 3 days for stabilization (to complete embryo development) and then floated in 1 l of deionized water (non-chlorinated) taken in metallic enamel-coated trays [18, 40]. Powdered dog biscuits and baker’s yeast pellets at a 3:2 ratio were given in adequate amounts for 2 days of larval food for the newly emerged larvae. The larval-rearing water was changed every 2 days for optimal larval growth and to reduce larval mortality. Trays were completely enclosed by wire mesh and maintained in optimal laboratory conditions as mentioned before, with routine monitoring.

A total of 1000 pupae were collected from the larval rearing tray, and each pupa was carefully placed in a 1.5-ml Eppendorf tube with 0.5 ml deionized water to avoid the chances of mating at the emergence of adults from the pupal stage [23]. Three tiny holes were made through the lid of each tube to facilitate the respiration of the pupa, and the tubes were maintained at 27 ± 1 °C and RH of 85 ± 5% with a photoperiod of 12:12 h (L:D) for 24 h. The sex of the emerging adults in the transparent Eppendorf tube was determined using a stereomicroscope (Nikon SMZ800, Japan). In contrast to the pilose (less hairy) antennae of females, the plumose (feather-like) antennae of males can be easily distinguished. By skipping the early pupae, we were able to ensure that we selected more female pupae than males.

### Blood-feeding versus mating

**Fig. 1:**
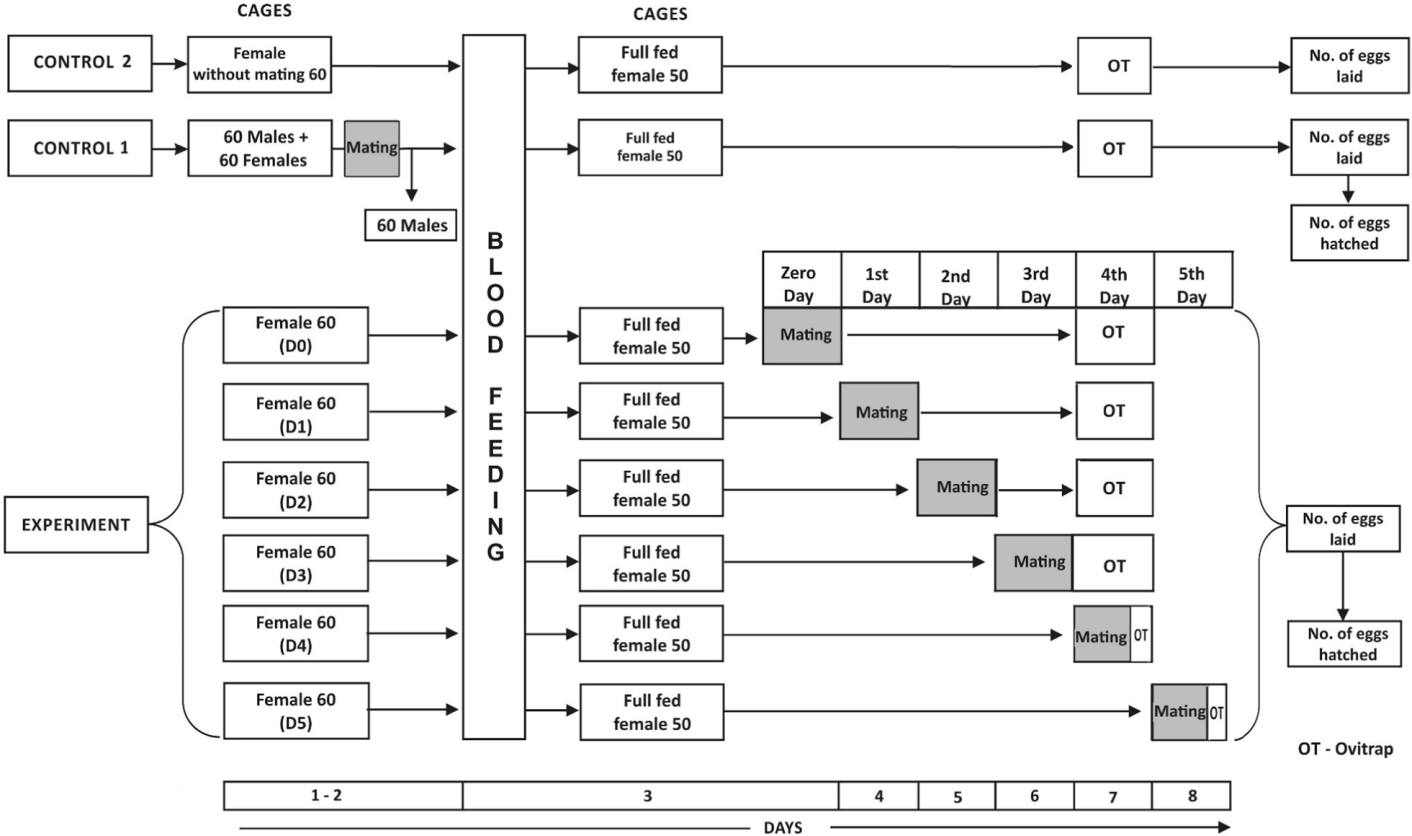
Schematic representation of the methodology followed

As we required only 480 females [60 females in each of eight cages (six for experiment + two for controls)] and 420 males [60 males in each of seven cages (six for experiment + one for control)], the excess males and females were kept as substitutes. Only active and healthy males and females were selected by discarding the inactive and sluggish individuals. Sixty emerged females were kept separately in each 60 × 60 × 60 cm cage labelled from day 0 (D0) to day 5 (D5) and control 1 and 2 as shown in the schematic diagram in Fig. 1, and all the males were kept together in one cage. Both adults were kept for 48 h with a cotton pad soaked in 10% glucose along with wet raisins as a sugar source to attain sexual maturity.

For control 1, a set of 60 sexually mature males and females were allowed to mate individually by releasing one male and one female in each of the 60 flat-bottom glass tubes (length 10 cm; diameter 2.5 cm) with a cotton plug. Generally, mating occurred within 10–15 min, with a few exceeding 30 min. Unmated pairs were noted and replaced by an equal number of other pairs to bring the total mated pairs to 60. After mating, the female from each tube was released back into a 60 × 60 × 60 cm cage, and the male was removed from each tube and discarded. The females were allowed to take a chicken blood meal for 2 h, after which they were examined for distended abdomen through the transparent cage for the status of individual feeding. Then, using a mechanical aspirator, the unfed and half-fed individuals were removed, keeping 50 fully fed females in the cage. A cotton pad soaked in 10% glucose along with wet raisins was given in each cage as a sugar source. On the fourth day of blood-feeding, an ovitrap was provided for oviposition. Since it has been established that for more than 90% of females, oviposition occurs within the first 30 min, the ovitraps were kept for 1 h to achieve maximum egg-laying. For control 2, 60 females were released into a 60 × 60 × 60 cm cage and allowed to take a chicken blood meal. Then the unfed and partially fed females were removed, leaving 50 fully fed females which were maintained as described for control 1.

For the experiment, six 60 × 60 × 60 cm cages were taken and marked as D0 to D5. Sixty sexually mature females were released in each cage and were allowed to take a chicken blood meal for 2 h. On the mating day, the engorged and partially/fully gravid females from cages D0 to D5 were taken separately from the cage and allowed to mate with individually released males as described for control 1 (Fig. 1). Utmost care was taken when collecting the fully fed females to avoid rupturing the distended abdomen. The mating was observed, and the glass tube with the mated female was marked and released back into the respective cage and maintained under the conditions noted earlier for colony maintenance. All the mated males were euthanized. The ovitrap was kept on the fourth day of blood-feeding for the cages marked D0 to D3 and collected after 1 h. For the cages marked D4 and D5, the ovitrap was kept immediately after mating on the same day, fourth, and fifth day, respectively, and collected after 1 h.

Plastic bowls (height 6 cm, upper diameter 7 cm, lower diameter 4.5 cm), half-filled with deionized water (200 ml), were used as ovitraps to collect the eggs. A filter paper (Whatman) strip was circularly placed around the inner wall of each bowl and kept half immersed in the water. The egg-laid papers were air-dried, and the number of eggs was counted under a stereomicroscope and recorded. The air-dried and 3-day-stabilized eggs in the Whatman filter papers were separately floated in deionized water and kept in enamel-coated metallic trays for hatching, as described above. The number of larvae hatched from the eggs in each cage were counted and recorded. In the subsequent days, the entire experiment was replicated twice with different cohorts of mosquitoes, and the mean value of the three experiments was used for the analysis.

### Statistical analysis

For statistical analysis, we first tested the normality of all data using the D’Agostino–Pearson normality test with the Chi-square distribution (right-tailed) to determine skewness (to quantify the asymmetry of the distribution) and kurtosis (to quantify the shape of the distribution). Since the normality assumptions were satisfied, we used one-way analysis of variance (ANOVA) to determine the mean variations within and between populations (SPSS ver. 15).

Then, the mean numbers of eggs laid and eggs hatched were represented in the Excel bar diagram with 5% error bars. The numbers of eggs laid and hatched by a single female were calculated from the total number of eggs laid and hatched and drawn as a line graph. Linear regression analysis was also conducted to determine the statistical significance of the study. The beta coefficient, *P*-value, and *R*-squared values were determined.

## Results

**Fig. 2:**
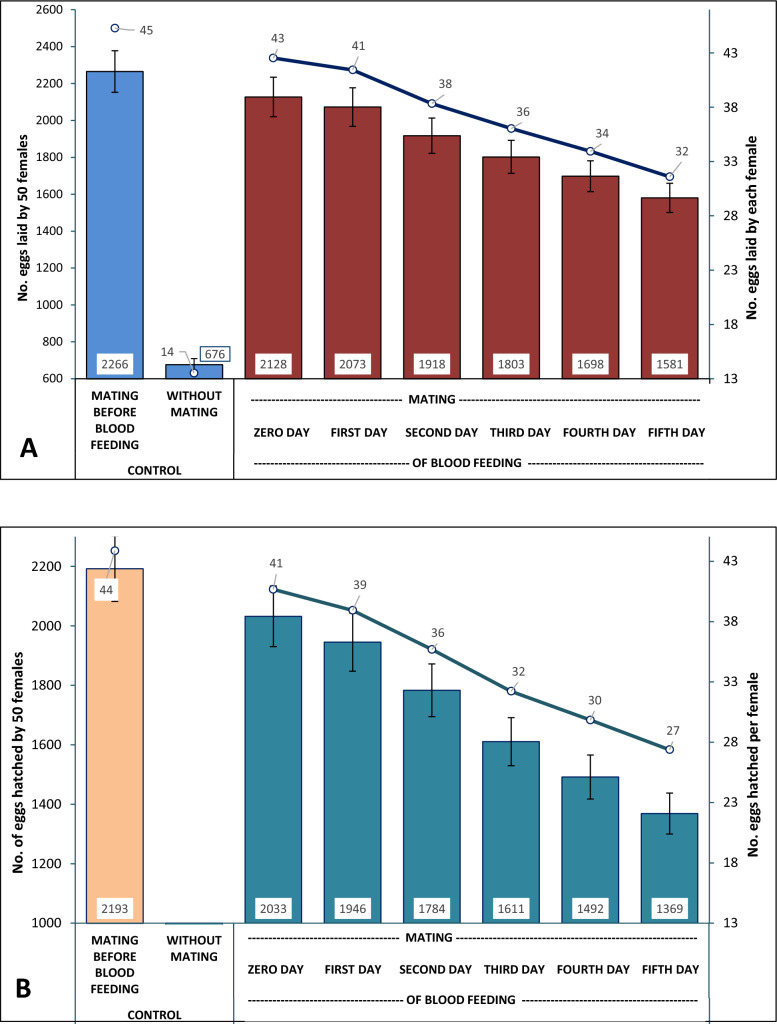
Mean number of eggs laid (**A**) and hatched (**B**) by the female *Ae. aegypti* mosquitoes mated on day 0 of blood-feeding to day 5 of blood-feeding with 5% error bars. Line graph shows the number of eggs laid or hatched by a single female

Out of 1000 pupae kept for emergence, a total of 523 females and 477 males emerged at the end of 24 h and were used as described in the previous section for one set of experiments. The mean of triplicate analysis showed that the females that mated before the blood meal in control 1 laid 2266 eggs, compared with control 2, where unmated females laid only 676 eggs, which is around 70% less than the mated females. In the experimental set, the cage marked D0, where the females were allowed to mate with males immediately after the blood meal, i.e. mating occurred on day 0 (day of blood-feeding), females laid an average of 2128 eggs, followed by 2073, 1918, 1803, 1698, and 1581 eggs by females mating on the first, second, third, fourth, and fifth days, respectively. The number of eggs laid decreased from day 0 to day 5 of mating when compared with the control, in which males were allowed to mate before blood-feeding (Fig. 2A).

The mean value of the number of eggs hatched showed that, in control 1, 2193 eggs were hatched (96.78%), which was the highest, where male mosquitoes were released and allowed to mate with a female before blood-feeding. None of the eggs laid by unmated females in control 2 hatched. In the experimental set, in the D0 cage, 2033 eggs were found hatching (95.54%), followed by 1946 (93.87%), 1784 (93.01%), 1611 (89.35%), 1492 (87.87%), and 1969 (86.59%) eggs found hatching in the cages where males were allowed to mate on the first, second, third, fourth, and fifth days of blood-feeding. Similar to the sequential reduction of the number of eggs laid, the number of eggs hatched also decreased from day 0 to day 5 of mating when compared with control 1 (Fig. 2B). It was also observed that when the gravid females from D3–D5 cages after mating on the third to fifth days were released back into the respective cages for oviposition, more than 90% of the females laid eggs within the first 30 min. On day 0, a single female laid 43 and hatched 41 eggs, followed by 41:39, 38:36, 36:32, 34:30, and 32:27, respectively, for the first to the fifth day of mating after blood-feeding. These numbers were 45:41 for control 2, and only 14 eggs were laid by a single female in control 1 without hatching.

**Fig. 3:**
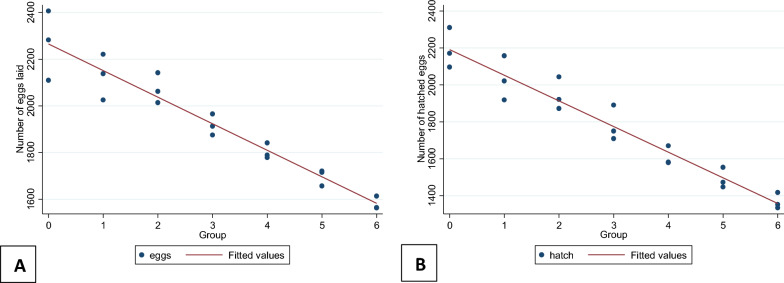
Linear regression analysis of number of eggs laid (**A**) and number of eggs hatched (**B**) by 50 female *Ae. aegypti*

For the number of eggs laid, the D’Agostino–Pearson test showed a significant difference from the normal distribution, and skewness was −0.05297 (*P* = 0.95) (potentially symmetrical). The excess kurtosis was −1.531 (*P* = 0.379) (potentially mesokurtic normal-like tails). The one-way ANOVA showed a significant difference in the number of eggs laid each day (*F* = 42.69, *df* = 17, *P* < 0.05) from day 0 to day 5 using three replicates (3 × 6 days). Similarly, for the number of eggs hatched, the D’Agostino–Pearson test showed a significant difference from the normal distribution. The skewness was 0.00855 (*P* = 0.992) (potentially symmetrical), and the excess kurtosis was −1.7022 (*P* = 0.328) (potentially mesokurtic normal-like tails). The one-way ANOVA showed a significant difference in the hatchability of each day’s eggs (*F* = 31.47, *df* = 17, *P* < 0.05) from day 0 to day 5 using three replicates (3 × 6 days). The linear regression analysis also showed that the number of eggs laid and the number of eggs hatched significantly decreased when days were increased, with *P* < 0.001 (Fig. 3). The beta coefficient and *R*^2^ values were 113.75 and 0.92, and 138.89 and 0.94, respectively, for the number of eggs laid and hatched.

## Discussion

This study demonstrated that mating can occur before or after blood-feeding. One of the major findings of the study is that mating a few minutes before egg-laying was sufficient to produce 1581 eggs (70% when compared with control) and fertilize 1369 eggs (85%), which is far higher than the 676 non-hatching (unfertilized) eggs (30%) laid by unmated females. This shows that although mating is not essential for producing eggs, even brief exposure to the semen and seminal fluids greatly enhanced the number of eggs and hatching efficiency, even if the mating occurred just before oviposition. The retention time for seminal fluids in the spermathecae of females seems to determine the number of eggs laid and hatched. The seminal fluid retention time was higher in control 1 compared with the females that mated on subsequent days, and hence, the females in control 1 that mated before blood-feeding produced more eggs and showed a higher percentage of hatching than those females that mated on subsequent days of blood-feeding. Females that mated on the fifth day may have had very little time to benefit from the seminal fluids other than fertilization; hence, only 1581 eggs were laid and only 1369 (86.59%) eggs hatched, compared with control 1, where 2266 eggs were laid and 2193 eggs hatched (96.78%). The females that mated on day 0 laid 2128 eggs and hatched 2033 eggs (95.54%). As the females in control 1 were not able to have mated with males and did not have the chance to benefit from the seminal fluids, their egg production was only 30% when compared with control 2. It was also clear that, even at the fully gravid stage, the spermathecae were capable of receiving sperm and seminal fluids from the males. In addition, it was demonstrated that after blood-feeding, if there are no males available to mate, the blood-fed female can delay mating up to minutes before oviposition and still produce viable progeny.

In control 1, females without mating also took blood meals and produced non-viable eggs, showing that mating is not a requirement for blood-feeding, which is similar to the view of Ramirez-Sanchez [28]. Although a few studies have reported post-mating responses (PMR) such as enhanced vectorial capacity [41], increased female host-seeking behaviours [42, 43], blood-feeding frequency [44], longevity [45], induction of transcriptional changes in female reproductive tract tissues [31], increased blood meal size [44], enhanced egg-laying rates [46, 47], and inhibition of re-mating [29, 30], the influence and outcome of mating before blood-feeding versus mating just before oviposition have not been reported elsewhere. Contrary to previous reports [36], which led to the assumption that blood-fed females have a lower success rate for copulation and/or take longer to copulate than their unfed counterparts, our observations clearly showed that the egg production was reduced by only 6% and viability was reduced by only 1.2% in comparing the females that mated before and after the blood meal. Wright and Venard [27] reported that there was no difference in the level of insemination between unfed and blood-fed females. This indicates that males had no preference either for females who had taken a blood meal or for those that had not. However, the decrease in the number of eggs laid and the number of eggs hatched when mating was delayed after blood-feeding indicates the importance of male factors in the fecundity of the females and the influence of semen and seminal fluids on the PMR. With respect to a single female, even a short course of exposure to semen and seminal fluids was sufficient to produce almost double the number of eggs, with 32 eggs produced by the female that mated just before (1 h) oviposition on the fifth day of blood-feeding, compared with only 14 eggs laid by the unmated female.

In light of these results, the present study may aid in gaining a clearer understanding of the reproductive biology of *Aedes* mosquitoes, which will further strengthen the knowledge in this field to develop novel control methods based on mating behaviour and reproduction. Understanding the mating behaviour in *Ae. aegypti* is of paramount importance for developing control techniques such as *Wolbachia*-based methods or genetic-based control methods [48]. Moreover, understanding the mating and blood-feeding behaviour in relation to the resting and gonotrophic cycles is important, as they can influence or even determine the impact of any intervention strategies. Reliable information on these behaviours in the vector population and on the behaviour of the human population is essential before the selection and implementation of any control method [15].

## Conclusions

This study demonstrates the possibility for mating of gravid *Ae. aegypti* mosquitoes minutes before oviposition and still producing viable eggs. Even brief exposure to the semen and seminal fluids of the male minutes before oviposition was found to be sufficient for female *Ae. aegypti* to produce viable eggs and also for producing a greater number of eggs when compared with unmated females.

## Data Availability

No datasets were generated or analysed during the current study.

## References

[1] Guzman MG, Harris E. Dengue. Lancet. 2015;385:453–65. 25230594 10.1016/S0140-6736(14)60572-9

[2] Alfonso-Parra C, Avila F. Molecular responses to the zika virus in mosquitoes. Pathogens. 2018;7:E49. 10.3390/pathogens7020049 PMC6027243 29751526

[3] Vega-Rúa A, Zouache K, Girod R, Failloux AB, Lourenço-de-Oliveira R. High level of vector competence of *Aedes aegypti* and *Ae. albopictus* from ten American countries as a crucial factor in the spread of Chikungunya virus. J Virol. 2014;88:6294–306. 24672026 10.1128/JVI.00370-14 PMC4093877

[4] Chippaux JP, Chippaux A. Yellow fever in Africa and the Americas: a historical and epidemiological perspective. J Venom Anim Toxins Incl Trop Dis. 2018;24:20. 30158957 10.1186/s40409-018-0162-y PMC6109282

[5] Bhatt S, Gething P, Brady O, Messina J, Farlow A, Moyes CL, et al. The global distribution and burden of dengue. Nature. 2013;496:504–7. 23563266 10.1038/nature12060 PMC3651993

[6] World Health Organization. Vector-borne diseases. 2020. https://www.who.int/news-room/fact-sheets/detail/vector-borne-diseases. Accessed 12 Aug 2023.

[7] Tuells J, Henao-Martínez AF, Franco-Paredes C. Yellow Fever: A Perennial Threat. Arch Med Res. 2022;53:649–57. 36404585 10.1016/j.arcmed.2022.10.005

[8] Kallas EG, Cintra MAT, Moreira JA, Patino EG, Braga PE, Tenório JCV, et al. Live, attenuated, tetravalent butantan-dengue vaccine in children and adults. N Engl J Med. 2024;390:397–408. 38294972 10.1056/NEJMoa2301790

[9] World Health Organization. Global strategy for dengue prevention and control 2012–2020. Geneva: World Health Organization; 2012.

[10] Manjarres-Suarez A, Olivero-Verbel J. Chemical control of *Aedes aegypti*: a historical perspective. Rev Costarric Salud Púb. 2013;22:68–75.

[11] Gan SJ, Leong YQ, Bin Barhanuddin MFH, Wong ST, Wong SF, Mak JW, et al. Dengue fever and insecticide resistance in Aedes mosquitoes in Southeast Asia: a review. Parasit Vectors. 2021;14:315. 10.1186/s13071-021-04785-4. 34112220 10.1186/s13071-021-04785-4 PMC8194039

[12] Spadar A, Collins E, Messenger LA, Clark TG, Campino S. Uncovering the genetic diversity in *Aedes aegypti* insecticide resistance genes through global comparative genomics. Sci Rep. 2024;14:13447. 38862628 10.1038/s41598-024-64007-6 PMC11166649

[13] Moyes CL, Vontas J, Martins AJ, Ng LC, Koou SY, Dusfour I, et al. Contemporary status of insecticide resistance in the major Aedes vectors of arboviruses infecting humans. PLoS Negl Trop Dis. 2017;11:e0005625. 28727779 10.1371/journal.pntd.0005625 PMC5518996

[14] Cox J, Mota J, Sukupolvi-Petty S, Diamond MS, Rico-Hesse R. Mosquito bite delivery of dengue virus enhances immunogenicity and pathogenesis in humanized mice. J Virol. 2012;86:7637–49. 22573866 10.1128/JVI.00534-12 PMC3416288

[15] Facchinelli L, Badolo A, McCall PJ. Biology and behaviour of *Aedes aegypti* in the human environment: opportunities for vector control of arbovirus transmission. Viruses. 2023;15:636. 36992346 10.3390/v15030636 PMC10053764

[16] Roth LM. A study of mosquito behaviour. An experimental laboratory study of the sexual behavior of *Aedes**aegypti* (Linnaeus). Am Midl Nat. 1948;40:265. 10.2307/2421604

[17] Rezende GL, Martins AJ, Gentile C, Farnesi LC, Pelajo-Machado M, Peixoto AA, et al. Embryonic desiccation resistance in *Aedes aegypti*: presumptive role of the chitinized serosal cuticle. BMC Dev Biol. 2008;13:82. 10.1186/1471-213X-8-82 PMC2561029 18789161

[18] Farnesi LC, Martins AJ, Valle D, Rezende GL. Embryonic development of *Aedes aegypti* (Diptera: Culicidae): influence of different constant temperatures. Mem Inst Oswaldo Cruz. 2009;104:124–6. 19274388 10.1590/S0074-02762009000100020

[19] Lima-Camara TN, Lima JBP, Bruno RV, et al. Effects of insemination and blood-feeding on locomotor activity of *Aedes**albopictus* and *Ae*. *aegypti* (Diptera: Culicidae) females under laboratory conditions. Parasit Vectors. 2014;7:304. 24990394 10.1186/1756-3305-7-304 PMC4094679

[20] Farnesi LC, Menna-Barreto RFS, Martins AJ, Valle D, Rezende GL. Physical features and chitin content of eggs from the mosquito vectors *Aedes aegypti*, *Anopheles aquasalis* and *Culex quinquefasciatus*: connection with distinct levels of resistance to desiccation. J Insect Physiol. 2015;83:43–52. 26514070 10.1016/j.jinsphys.2015.10.006

[21] Farnesi LC, Vargas HCM, Valle D, Rezende GL. Darker eggs of mosquitoes resist more to dry conditions: melanin enhances serosal cuticle contribution in egg resistance to desiccation in Aedes, Anopheles and Culex vectors. PLoS Negl Trop Dis. 2017;11:10. 10.1371/journal.pntd.0006063 PMC5679640 29084225

[22] Farnesi LC, Carvalho FD, Lacerda APC, Moreira LA, Bruno RV. The influence of different sources of blood meals on the physiology of *Aedes**aegypti* harboring *Wolbachia *wMel: mouse blood as an alternative for mosquito rearing. Parasit Vectors. 2021;14:21. 33407798 10.1186/s13071-020-04465-9 PMC7787405

[23] Dieng H, Satho T, Abang F, Wydiamala E, Kassim NF, Hashim NA, et al. Sex before or after blood feeding: mating activities of *Aedes aegypti* males under conditions of different densities and female blood feeding opportunities. J Asia Pac Entomol. 2019;22:274–80. 10.1016/j.aspen.2018.12.025

[24] Cabrera M, Jaffe K. An aggregation pheromone modulates lekking behavior in the vector mosquito *Aedes aegypti* (Diptera: Culicidae). J Am Mosq Control Assoc. 2007;23:1–10. 17536361 10.2987/8756-971X(2007)23[1:AAPMLB]2.0.CO;2

[25] Ponlawat A, Harrington LC. Factors associated with male mating success of the dengue vector mosquito, *Aedes aegypti*. Am J Trop Med Hyg. 2009;80:395–400. 19270288 10.4269/ajtmh.2009.80.395

[26] O’Meara GF. Gonotrophic Interactions in Mosquitoes: Kicking the Blood-Feeding Habit. Fla Entomol. 1985;68:122–33. 10.2307/3494335

[27] Wright JE, Venard CE. The influence of a blood meal on copulation in *Aedes triseriatus* (Diptera: Culicidae). Ann Entomol Soc Am. 1967;60:861–2. 10.1093/aesa/60.4.861a

[28] Ramírez-Sánchez LF, Hernández BJ, Guzmán PA, Alfonso-Parra C, Avila FW. The effects of female age on blood-feeding, insemination, sperm storage, and fertility in the dengue vector mosquito *Aedes aegypti* (Diptera: Culicidae). J Insect Physiol. 2023;150:104570. 37806552 10.1016/j.jinsphys.2023.104570

[29] Degner EC, Harrington LC. Polyandry depends on post-mating time interval in the dengue vector *Aedes aegypti*. Am J Trop Med Hyg. 2016;94:780–5. 26880776 10.4269/ajtmh.15-0893 PMC4824218

[30] Helinski MEH, Deewatthanawong P, Sirot LK, Wolfner MF, Harrington LC. Duration and dose-dependency of female sexual receptivity responses to seminal fluid proteins in *Aedes albopictus* and *Ae. aegypti* mosquitoes. J Insect Physiol. 2012;58:1307–13. 22796224 10.1016/j.jinsphys.2012.07.003 PMC3438290

[31] Alfonso-Parra C, Ahmed-Braimah YH, Degner EC, Avila FW, Villarreal SM, Pleiss JA, et al. Mating-induced transcriptome changes in the reproductive tract of female *Aedes aegypti*. PLoS Negl Trop Dis. 2016;10:e0004451. 26901677 10.1371/journal.pntd.0004451 PMC4764262

[32] Camargo C, Ahmed-Braimah YH, Amaro IA, Harrington LC, Wolfner MF, Avila FW. Mating and blood-feeding induce transcriptome changes in the spermathecae of the yellow fever mosquito *Aedes aegypti*. Sci Rep. 2020;10:14899. 32913240 10.1038/s41598-020-71904-z PMC7484758

[33] Pascini TV, Ramalho-Ortig˜ao M, Ribeiro JM, Jacobs-Lorena M, Martins GF. Transcriptional profiling and physiological roles of *Aedes aegypti* spermathecal-related genes. BMC Genom. 2020;21:143. 10.1186/s12864-020-6543-y PMC7011475 32041546

[34] Shaw WR, Teodori E, Mitchell SN, Baldini F, Gabrieli P, Rogers DW, et al. Mating activates the heme peroxidase HPX15 in the sperm storage organ to ensure fertility in *Anopheles gambiae*. Proc Natl Acad Sci. 2014;111:5854–9. 24711401 10.1073/pnas.1401715111 PMC4000814

[35] Christophers SR. *Aedes**aegypti* (L.) the Yellow Fever Mosquito Its life history, bionomics, and structure. New York: Cambridge University Press; 1960.

[36] Oliva CF, Damiens D, Benedict MQ. Male reproductive biology of *Aedes* mosquitoes. Acta Trop. 2014;132:S12–9. 24308996 10.1016/j.actatropica.2013.11.021

[37] Degner EC, Harrington LC. Polyandry depends on post mating time interval in the dengue vector *Aedes aegypti*. Am J Trop Med Hyg. 2016;94:780–5. 26880776 10.4269/ajtmh.15-0893 PMC4824218

[38] Duvall LB, Basrur NS, Molina H, Mc Meniman CJ, Vosshall LB. A peptide signalling system that rapidly enforces paternity in the *Aedes aegypti* mosquito. Curr Biol. 2017;27:3734–42. 29174895 10.1016/j.cub.2017.10.074 PMC5730346

[39] Zheng ML, Zhang DJ, Damiens DD, Lees RS, Gilles JR. Standard operating procedures for standardized mass rearing of the dengue and chikungunya vectors *Aedes aegypti* and *Aedes albopictus* (Diptera: Culicidae)–II–egg storage and hatching. Parasit Vectors. 2015;8:348. 26112698 10.1186/s13071-015-0951-x PMC4485872

[40] Clemons A, Mori A, Haugen M, Severson DW, Duman-Scheel M. Culturing and egg collection of *Aedes aegypti*. Cold Spring Harb Protoc. 2010;2010(10):pdb.prot5507. 10.1101/pdb.prot5507. 20889704 10.1101/pdb.prot5507 PMC2966317

[41] Kramer LD, Ciota AT. Dissecting vectorial capacity for mosquito-borne viruses. Curr Opin Virol. 2015;15:112–8. 26569343 10.1016/j.coviro.2015.10.003 PMC4688158

[42] Klowden MJ. The check is in the male: male mosquitoes affect female physiology and behavior. J Am Mosq Control Assoc. 1999;15:213–20. 10412116

[43] Lee JJ, Klowden MJ. A male accessory gland protein that modulates female mosquito (Diptera: Culicidae) host-seeking behavior. J Am Mosq Control Assoc. 1999;15:4–7. 10342262

[44] Villarreal SM, Pitcher S, Helinski MEH, Johnson L, Wolfner MF, Harrington LC. Male contributions during mating increase female survival in the disease vector mosquito *Aedes aegypti*. J Insect Physiol. 2018;108:1–9. 29729859 10.1016/j.jinsphys.2018.05.001 PMC5988987

[45] Helinski ME, Harrington LC. Male mating history and body size influence female fecundity and longevity of the dengue vector *Aedes**aegypti*. J Med Entomol. 2011;48:202–11. 21485355 10.1603/ME10071 PMC4182911

[46] Judson CL. Feeding and oviposition behavior in the mosquito *Aedes**aegypti* (L.) I. Preliminary studies of physiological control mechanisms. Biol Bull. 1967;133:369–78. 6062263 10.2307/1539832

[47] Hiss EA, Fuchs MS. The effect of matrone on oviposition in the mosquito, *Aedes**aegypti*. J Insect Physiol. 1972;18:2217–27. 4653439 10.1016/0022-1910(72)90250-8

[48] Carvalho DO, McKemey AR, Garziera L, Lacroix R, Donnelly CA, Alphey L, et al. Suppression of a field population of *Aedes aegypti* in Brazil by sustained release of transgenic male mosquitoes. PLOS Negl Trop Dis. 2015;9:e0003864. 26135160 10.1371/journal.pntd.0003864 PMC4489809

